# Therapeutic Efficacy of Topically Applied Antioxidant Medicinal Plant Extracts in a Mouse Model of Experimental Dry Eye

**DOI:** 10.1155/2016/4727415

**Published:** 2016-05-26

**Authors:** Won Choi, Jee Bum Lee, Lian Cui, Ying Li, Zhengri Li, Ji Suk Choi, Hyo Seok Lee, Kyung Chul Yoon

**Affiliations:** ^1^Department of Ophthalmology and Center for Creative Biomedical Scientists, Chonnam National University Medical School and Hospital, Gwangju 61469, Republic of Korea; ^2^Department of Dermatology, Chonnam National University Medical School and Hospital, Gwangju 61469, Republic of Korea; ^3^Eye Institute of Xiamen University, Fujian Provincial Key Laboratory of Ophthalmology and Visual Science, Xiamen, Fujian 361100, China

## Abstract

*Purpose*. To investigate the therapeutic effects of topical administration of antioxidant medicinal plant extracts in a mouse model of experimental dry eye (EDE).* Methods*. Eye drops containing balanced salt solution (BSS) or 0.001%, 0.01%, and 0.1% extracts were applied for the treatment of EDE. Tear volume, tear film break-up time (BUT), and corneal fluorescein staining scores were measured 10 days after desiccating stress. In addition, we evaluated the levels of interleukin- (IL-) 1*β*, tumor necrosis factor- (TNF-) *α*, IL-6, interferon- (IFN-) *γ*, and IFN-*γ* associated chemokines, percentage of CD4+C-X-C chemokine receptor type 3 positive (CXCR3+) T cells, goblet cell density, number of 4-hydroxy-2-nonenal (4-HNE) positive cells, and extracellular reactive oxygen species (ROS) production.* Results*. Compared to the EDE and BSS control groups, the mice treated with topical application of the 0.1% extract showed significant improvements in all clinical parameters, IL-1*β*, IL-6, TNF-*α*, and IFN-*γ* levels, percentage of CD4+CXCR3+ T cells, goblet cell density, number of 4-HNE-positive cells, and extracellular ROS production (*P* < 0.05).* Conclusions*. Topical application of 0.1% medicinal plant extracts improved clinical signs, decreased inflammation, and ameliorated oxidative stress marker and ROS production on the ocular surface of the EDE model mice.

## 1. Introduction

Dry eye disease (DED) is a common ocular surface disorder described as a disorder of the tear film caused by reduced tear production, poor tear quality, or excessive tear evaporation [1]. This disorder is associated with symptoms of ocular discomfort such as dryness, irritation, foreign body sensation, redness, and itching [2]. It affects the lives of millions of people, with a prevalence as high as 14.5%, which continues to rise [3]. Abundant evidence suggests that increased osmolarity of the tear film and T cell mediated inflammation of the ocular surface are involved in the pathogenesis of the disease [4–7]. However, the precise pathogenesis of DED is not entirely understood. Recently, it was recognized that oxidative stress has a prominent role in the development of DED [8–11].

Studies, both* in vivo* and* in vitro*, have demonstrated that reactive oxygen species (ROS) overproduction and oxidative stress are an underlying mechanism of ocular surface diseases such as DED [12–14]. A close relationship between lipid peroxidation-related membrane damage, protein oxidation, ROS production, and inflammatory processes has been observed in patients and animal models of DED [8, 9, 15–20]. Therefore, the evaluation of the effects of oxidative stress on the ocular surface in dry eye could lead to a new approach about DED therapy that is based on the reduction of inflammation by the inhibition of this process. Molecules such as alpha-lipoic acid, xanthan gum, blueberry component, omega-3 essential fatty acids, mineral oil, L-carnitine, and selenium have been proven to prevent or treat DED in which oxidative stress-induced inflammation plays an important role in the disease pathogenesis [21–27].

In our previous study, we established the efficacy of a mixture of ethyl alcohol (EtOH) extracts of medicinal plants, including* Schizonepeta tenuifolia* var.* japonica Kitagawa, Angelica dahurica* Bentham et Hooker,* Rehmannia glutinosa Liboschitz* var.* purpurea Makino*, and* Cassia tora* L. which have antioxidant and anti-inflammatory properties in protecting human corneal epithelial cells against oxidative stress induced by irradiation from a short wavelength light emitting diode (LED) [28]. Based on this experiment, we also evaluated the clinical effect of glasses containing these antioxidant extracts by using a prospective, multicenter, double-blind, randomized, placebo-controlled trial and demonstrated their effectiveness in improving both subjective and objective parameters in patients with DED [29].

The antioxidant effects of medicinal plant extracts have been proven in human corneal epithelial cells* in vitro* and patients with DED, as we previously described. However, the antioxidant effects of medicinal plant extracts have not been elucidated using* in vivo* experimental dry eye (EDE) models. Therefore, we investigated the role of these extracts on clinical parameters, oxidative stress, and inflammatory markers in a mouse model of EDE in order to support topical use in ophthalmic formulations for the treatment of DED.

## 2. Methods

### 2.1. Mouse Model of Dry Eye

The research protocol was approved by the Chonnam National University Medical School Research Institutional Animal Care and Use Committee. All the animals were treated according to the standards of the Association for Research in Vision and Ophthalmology Statement for the Use of Animals in Ophthalmic and Vision Research.

This study used 6- to 8-week-old C57BL/6 female mice in the experiments. The EDE model was induced by subcutaneously injecting the mice with 0.5 mg/0.2 mL scopolamine hydrobromide (Sigma-Aldrich, St. Louis, MO, USA) four times a day (9 am, 1 pm, 5 pm, and 9 pm) with exposure to an air draft and 30% ambient humidity [24, 25]. During these experiments, the behavior, food, and water intake of the animals were not restricted.

### 2.2. Materials

The mixed medicinal plants used in these experiments were prepared as we previously described and consisted of* Schizonepeta tenuifolia *var.* japonica Kitagawa, Angelica dahurica *Bentham et Hooker*, Rehmannia glutinosa Liboschitz *var.* purpurea Makino, and Cassia tora *L. [28, 29]. All the plants materials were air-dried and powdered using a plant grinding machine. A 100 g sample of the powder was extracted with 1000 mL of 95% aqueous EtOH with continuous shaking for 12 hours at room temperature and then filtered using Whatman Number 4 qualitative filter paper. This procedure was repeated three times to ensure a complete extraction of the plant material. The extracts were concentrated by evaporation under reduced pressure in a rotary evaporator at 40°C and then freeze-dried [28]. The antioxidant medicinal plant EtOH extracts were diluted with balanced salt solution (BSS, Alcon, Fort Worth, TX, USA).

### 2.3. Experimental Designs and Procedures

The mice were randomly assigned to six groups, which were treated with the topical extracts as follows: (1) untreated control (UT), not exposed to desiccating stress or treated topically; (2) EDE control, exposed to desiccating stress, however, receiving no eye drops; (3) BSS control, EDE mice treated with BSS; (4) EDE mice treated with 0.001% EtOH extract; (5) EDE mice treated with 0.01% EtOH extract; and (6) EDE mice treated with 0.1% EtOH extract. Two microliters of the eye drops was applied topically to both eyes of unanesthetized mice three times a day until they were euthanized. The tear volume, tear film break-up time (BUT), and corneal fluorescein staining scores were measured 10 days after treatment. After the evaluation of the clinical parameters, the mice were euthanized, and multiplex immunobead assay, flow cytometry, histology, immunohistochemistry, and 2′,7′-dichlorodihydrofluorescein diacetate (DCFDA) assays were performed. Each group consisted of 10 animals, and the experiments were performed on two independent sets of mice.

### 2.4. Tear Volume Measurements

The tear volume of the mice was measured using phenol red-impregnated cotton threads (Zone-Quick, Oasis, Glendora, CA, USA) as previously mentioned [24, 30]. Briefly, the cotton threads were applied to the lateral canthus for 20 seconds and the tear volume, which was expressed in millimeters of the thread that was turned red by the tears, was measured using a microscope (SMZ 1500, Nikon, Melville, NY, USA). A standard curve was constructed to convert distance to volume.

### 2.5. Evaluation of Tear Film BUT and Corneal Staining Scores

After instilling 1 *μ*L of 1% sodium fluorescein into the inferior conjunctival sac using a micropipette, the mouse was allowed to blink thrice and then the tear film BUT was recorded in seconds in a masked fashion using a slit lamp biomicroscopy (BQ-900, Haag-Streit, Bern, Switzerland) under cobalt blue light. After the measurement of the tear film BUT was performed, the punctate staining of the corneal surface was evaluated. Each cornea was divided into four quadrants that were scored individually. The intensity of corneal staining was calculated using a 4-point scale (0–4): 0, absence of staining; 1, slightly punctate staining < 30 spots; 2, punctate staining > 30 spots, but not diffuse; 3, severe diffuse staining but no positive plaque; and 4, positive fluorescein plaque. The scores of the four areas of each mouse were summed to obtain the final score, ranging from 0 to 16 [31].

### 2.6. Multiplex Immunobead Assay

A multiplex immunobead assay (Luminex 200, Luminex Corp., Austin, TX, USA) was used to measure the concentrations of interleukin- (IL-) 1*β*, IL-6, tumor necrosis factor- (TNF-) *α*, interferon- (IFN-) *γ*, monokine induced by IFN-*γ* (MIG), and IFN-*γ*-induced protein- (IP-) 10 in the conjunctiva, as previously stated [32]. In summary, the conjunctival tissues were collected (four eyes per group) and pooled in lysis buffer containing protease inhibitors for 30 min. The cell extracts were centrifuged at 12,000 rpm for 15 minutes at 4°C, and the supernatants were stored at −70°C until being analyzed. The total protein concentration of the supernatants was determined, and 25 *μ*L of total protein of each sample was pipetted into assay plate wells. The supernatants were then added to the wells containing the appropriate cytokine bead mixture that included mouse monoclonal antibodies specific for the cytokines and chemokines for 60 min. After three washes, the plate was incubated for 30 min in the dark at room temperature using a biotinylated detection antibody. The reactions were detected using an analysis system (xPONET, Austin, TX) after the addition of streptavidin-phycoerythrin. The tissue concentrations of the cytokines and chemokines were calculated from the standard curves of known concentrations of recombinant mouse cytokines.

### 2.7. Flow Cytometry

Flow cytometry was performed to quantify the number of CD4+C-X-C chemokine receptor type 3 positive (CXCR3+) T cells in the conjunctiva as previously described [31]. The conjunctival tissues (four eyes per group) were harvested, dipped in phosphate-buffered saline (PBS), teased apart using scissors, and then shaken at 37°C for 60 minutes in the presence of 0.5 mg/mL collagenase type D (Roche Applied Science, Indianapolis, IN, USA). After incubation, the tissues were disrupted by grinding using a syringe plunger and passed through a cell strainer with a pore size of 100 mm. The cells were centrifuged at 1500 rpm for 7 minutes and then resuspended in PBS with 1% bovine serum albumin (BSA). After washing, the samples were incubated with the fluorescein-conjugated anti-CD4 antibody (BD Biosciences, San Jose, CA, USA), phycoerythrin-conjugated anti-CXCR3 antibody (clone 173, BD Biosciences), and isotype control antibody at 37°C for 30 minutes. The phycoerythrin-conjugated rat IgG isotype (BD Biosciences) was used as the control. The number of CD4+CXCR3+ T cells was counted using a fluorescence-activated cell sorting Calibur cytometer with the CellQuest software (both from BD Bioscience, Fullerton, CA, USA).

### 2.8. Histology

The eyes and adnexa (four eyes per group) were surgically excised, fixed in 4% paraformaldehyde, and embedded in paraffin. The tissue was then cut into 6 *μ*m sections, which were stained with a periodic acid-Schiff (PAS) reagent and then examined and photographed using a microscope (Olympus, Tokyo, Japan) equipped with a digital camera. The goblet cell density in the superior and inferior conjunctiva was measured in three sections from each eye using the image analysis software (Medial Cybernetics, Silver Spring, MD, USA) and was expressed as the number of goblet cells per 100 *μ*m.

### 2.9. Immunohistochemistry

The eyes and adnexa (four eyes per group) were surgically excised, fixed in 4% buffered paraformaldehyde solution overnight at 4°C, and paraffin-embedded. Then, the oxidative stress-induced lipid peroxidation marker was evaluated by immunohistochemically detecting 4-hydroxy-2-nonenal (4-HNE) in the conjunctiva. Six-micrometer sections were cut from the paraffin wax blocks, mounted on precoated glass slides, deparaffinized, and rehydrated. Then, 0.3% hydrogen peroxide (H_2_O_2_) and 1% serum both in PBS were sequentially applied to conjunctival sections, which were then incubated with mouse anti-4-HNE monoclonal antibody (JaICA, Shizuoka, Japan) at a concentration of 25 *μ*g/mL for 1 h at room temperature. After washing the sections, they were incubated with the rabbit anti-mouse IgG secondary antibodies. Then, the tissue sections were incubated with avidin-peroxidase, followed by the 3,3′-diaminobenzidine peroxidase substrate, and then counterstained with Mayer's hematoxylin. The number of positively stained cells for 4-HNE per 100 *μ*m was counted.

### 2.10. Measurement of ROS Production

The level of extracellular ROS production was measured using a DCFDA kit (Invitrogen, Carlsbad, CA, USA) according to the manufacturer's protocol, as previously mentioned [28]. In summary, surgically excised mice conjunctiva (four eyes per group) was washed with PBS and 10 *μ*M DCFDA and then incubated at 37°C for 20 minutes. To determine the extracellular ROS production, the cell pellet was analyzed using a FACSCalibur flow cytometer at excitation and emission wavelengths of 488 and 530 nm, respectively. The data analysis was based on 10,000 detected events using flow and image cytometry analysis software. The relative changes in DCFDA fluorescence were expressed as the fold increase compared to that in the UT group.

### 2.11. Statistical Analysis

The data are presented as mean ± standard deviation (SD). The statistical package for the social sciences (SPSS) software version 21.0 (SPSS, Chicago, IL, USA) was used for the statistical analyses. The differences in the tear volume, tear film BUT, and fluorescein staining scores between the groups were evaluated by using a one-way analysis of variance (ANOVA) test with Tukey's* post hoc* analysis. The Kruskal-Wallis and Mann-Whitney *U* test were used to compare the cytokine and chemokine levels, goblet and 4-HNE-positive cell densities, and flow cytometry differences between the groups. A *P* value < 0.05 was considered statistically significant.

## 3. Results

### 3.1. Tear Volume

**Figure 1:**
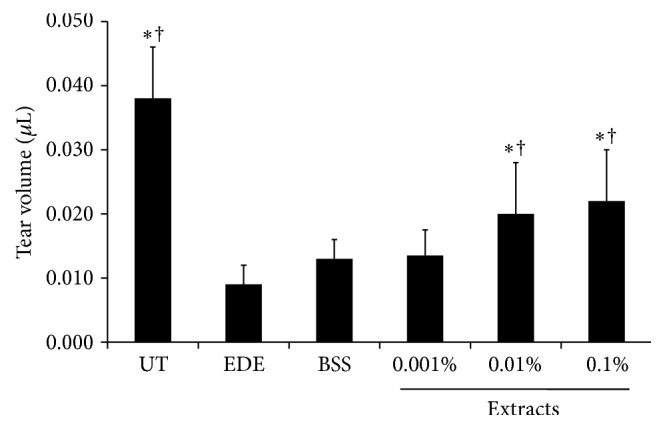
Mean tear volumes of untreated control (UT), experimental dry eye (EDE), balanced salt solution (BSS), and 0.001%, 0.01%, and 0.1% extract-treated groups 10 days after desiccating stress. The 0.01% and 0.1% extract-treated groups showed significant increase in tear volume compared to the EDE and BSS groups. ^*∗*^ *P* < 0.05 and ^†^ *P* < 0.05 compared with EDE and BSS-treated groups, respectively.

Ten days after the induction of EDE in the mice, the mean tear volumes were 0.038 ± 0.008 *μ*L, 0.009 ± 0.003 mL, and 0.013 ± 0.003 mL in the UT, EDE, and BSS-treated groups, respectively (*P* = 0.10 compared with the EDE group). Furthermore, the values were 0.013 ± 0.004 *μ*L in the 0.001% extract-treated group (*P* = 0.06 compared with the EDE group; *P* = 0.79 compared with the BSS-treated group), 0.020 ± 0.008 *μ*L in the 0.01% extract-treated group (*P* < 0.01 compared with the EDE and BSS-treated groups), and 0.022 ± 0.008 *μ*L in the 0.1% extract-treated group (*P* < 0.01 compared with the EDE and BSS-treated groups) (Figure 1). There were no differences in the tear volumes between the groups at baseline (data not shown).

### 3.2. Tear Film BUT

**Figure 2:**
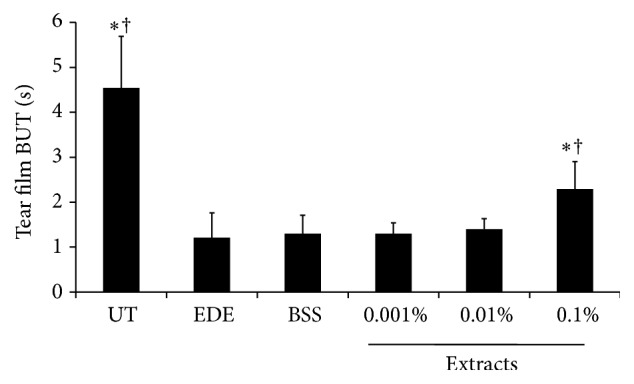
Tear film break-up time (BUT) of untreated control (UT), experimental dry eye (EDE), balanced salt solution (BSS), and 0.001%, 0.01%, and 0.1% extract-treated groups 10 days after desiccating stress. The 0.1% extract-treated group showed significant improvement in tear film BUT compared to the EDE and BSS groups. ^*∗*^ *P* < 0.05 and ^†^ *P* < 0.05 compared with EDE and BSS-treated groups, respectively.

At baseline, the mean corneal fluorescein staining scores in the six different groups showed no statistically significant differences (data not shown). After 10 days of desiccating stress, the tear film BUT was significantly shorter in the EDE group (1.21 ± 0.55 s) than it was in the UT group (4.54 ± 1.15 s, *P* < 0.01). The 0.1% extract-treated (2.29 ± 0.61 s) group showed a significant increase in tear film BUT compared to that in the EDE and BSS-treated groups (all *P* < 0.01), while the BSS and 0.001%, and 0.01% extract-treated groups (1.30 ± 0.41, 1.30 ± 0.24, and 1.40 ± 0.23 s, resp.) showed no significant improvement compared with the EDE group (*P* = 0.98, *P* = 0.98, and *P* = 0.76, resp.) (Figure 2).

### 3.3. Corneal Fluorescein Staining

**Figure 3:**
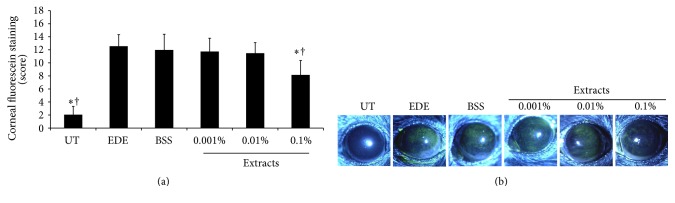
Mean corneal fluorescein staining scores (a) and representative figures (b) of untreated control (UT), experimental dry eye (EDE), balanced salt solution (BSS), and 0.001%, 0.01%, and 0.1% extract-treated groups 10 days after desiccating stress. The 0.1% extract-treated group showed significant decrease in corneal fluorescein staining compared with the EDE and BSS groups. ^*∗*^ *P* < 0.05 and ^†^ *P* < 0.05 compared with EDE and BSS-treated groups, respectively.

On day 10, the corneal fluorescein staining scores significantly increased in the EDE group (12.55 ± 1.77) compared with the UT group (2.05 ± 1.26, *P* < 0.01). The mean corneal fluorescein scores of the four treatment groups were 11.98 ± 2.41, 11.73 ± 2.05, 11.48 ± 1.62, and 8.15 ± 2.21 for the BSS and 0.001%, 0.01%, and 0.1% extract-treated groups, respectively. The 0.1% extract-treated group showed significant increases in corneal fluorescein score compared to those in the EDE and BSS groups (all *P* < 0.01) (Figure 3). There were no statistically significant differences in the mean corneal fluorescein staining scores of the six different groups at baseline (data not shown).

### 3.4. Inflammatory Cytokine and Chemokine Levels in Conjunctival Tissue

**Figure 4:**
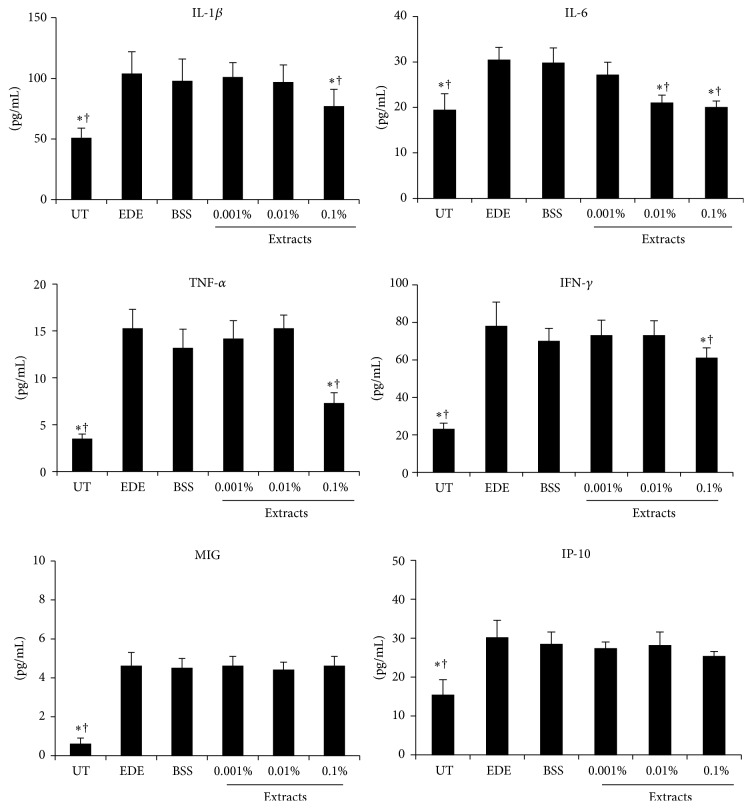
Concentrations of interleukin- (IL-) 1*β*, IL-6, tumor necrosis factor- (TNF-) *α*, interferon- (IFN-) *γ*, monokine induced by IFN-*γ* (MIG), and IFN-*γ*-induced protein- (IP-) 10 in untreated control (UT), experimental dry eye (EDE), balanced salt solution (BSS), and 0.001%, 0.01%, and 0.1% extract-treated groups 10 days after desiccating stress. ^*∗*^ *P* < 0.05 and ^†^ *P* < 0.05 compared with EDE and BSS-treated groups, respectively.

The concentrations of IL-1*β*, IL-6, TNF-*α*, and IFN-*γ* in the conjunctiva increased 10 days after induction of EDE (all *P* < 0.01) compared with the UT group, and these elevations significantly decreased after instillation of 0.1% extracts for 10 days (all *P* < 0.01). In addition, the IL-6 concentrations were significantly decreased 10 days after instillation of 0.01% extract compared with the EDE and BSS-treated groups (all *P* < 0.01). However, the MIG and IP-10 levels did not show significant differences in all treatment groups compared with the EDE group at day 10. No differences in the inflammatory cytokine and chemokine levels were observed among the EDE, BSS, and 0.001% extract-treated groups (all *P* > 0.05). The results of the inflammatory cytokine and chemokine levels determination in the conjunctival tissues are shown in Figure 4.

### 3.5. Flow Cytometric Analysis

**Figure 5:**
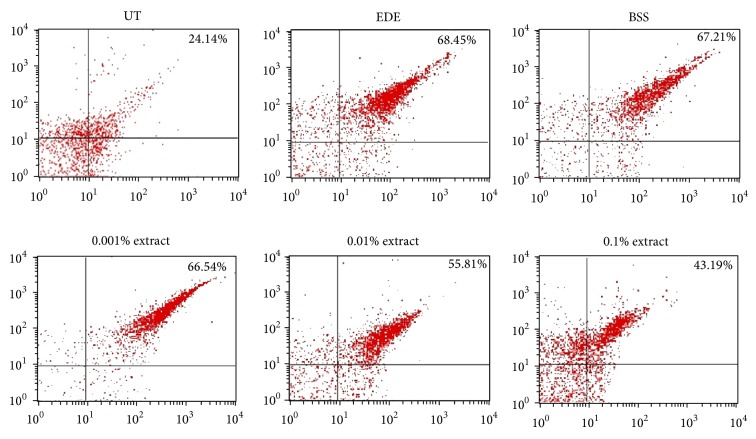
Representative histograms showing percentage of CD4+CXCR3+ T cells in conjunctiva in untreated control (UT), experimental dry eye (EDE), balanced salt solution (BSS), and 0.001%, 0.01%, and 0.1% antioxidant medicinal plant extract-treated groups 10 days after desiccating stress.

The histograms of the percentages of CD4+CXCR3+ T cells of representative samples from the UT, EDE, BSS, and 0.001%, 0.01%, and 0.1% extract-treated groups are shown in Figure 5. The number of CD4+CXCR3+ T cells significantly decreased in the 0.1% extract-treated group compared with the EDE and BSS-treated groups (all *P* < 0.01). The respective percentages of the CD4+CXCR3+ T cells were 22.54 ± 3.74%, 65.76 ± 10.47%, 64.82 ± 7.50%, 65.85 ± 7.14%, 54.85 ± 12.14%, and 43.38% ± 9.49% for the UT, EDE, BSS, and 0.001%, 0.01%, and 0.1% extract-treated groups, respectively.

### 3.6. Conjunctival Goblet Cell Density

**Figure 6:**
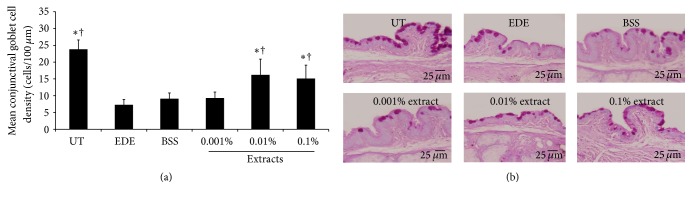
Mean number of conjunctival goblet cells (a) and representative figures (b) of untreated control (UT), experimental dry eye (EDE), balanced salt solution (BSS), and 0.001%, 0.01%, and 0.1% extract-treated groups 10 days after desiccating stress. The 0.01% and 0.1% extract-treated groups show a significantly higher number of goblet cells than the EDE and BSS groups. ^*∗*^ *P* < 0.05 and ^†^ *P* < 0.05 compared with EDE and BSS-treated groups, respectively.

Ten days after the mice were subjected to desiccating stress, the mean density of conjunctival goblet cells significantly decreased in the EDE group (7.25 ± 1.58 cells/100 *μ*m) compared with the UT group (23.88 ± 2.75 cells/100 *μ*m, *P* < 0.01). The mean goblet cell densities of the 0.01% (15.38 ± 4.00 cells/100 *μ*m) and 0.1% (15.13 ± 4.02 cells/100 *μ*m) extract-treated groups showed significant increases compared with EDE and BSS-treated (9.13 ± 1.73 cells/100 *μ*m) groups (all *P* < 0.01) (Figure 6).

### 3.7. 4-HNE Expression in Conjunctival Tissue

**Figure 7:**
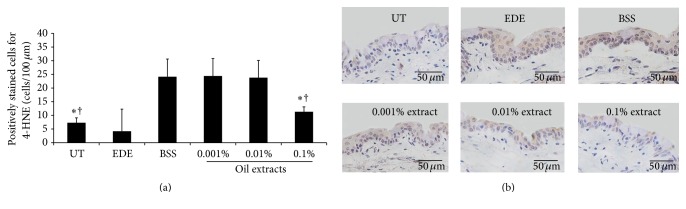
The number of positively stained cells for 4-hydroxy-2-nonenal (4-HNE) in the conjunctiva (a) and representative specimens (b) of untreated control (UT), experimental dry eye (EDE), balanced salt solution (BSS), and 0.001%, 0.01%, and 0.1% extract-treated groups 10 days after desiccating stress. ^*∗*^ *P* < 0.05 and ^†^ *P* < 0.05 compared with EDE and BSS-treated groups, respectively.

On day 10 after desiccating stress, the mean density of the conjunctival 4-HNE-positive cells was 7.38 ± 1.77 cells/100 *μ*m in the UT group, 24.25 ± 8.10 cells/100 *μ*m in the EDE group, 24.13 ± 6.53 cells/100 *μ*m in the BSS-treated group, 24.38 ± 6.41 cells/100 *μ*m in the 0.001% extract-treated group, 23.88 ± 6.33 cells/100 *μ*m in the 0.01% extract-treated group, and 11.38 ± 1.77 cells/100 *μ*m in the 0.1% extract-treated groups. Treatment with the topical 0.1% extract significantly decreased the number of positively stained cells for 4-HNE compared with EDE and BSS-treated groups (all *P* < 0.01) (Figure 7).

### 3.8. Generation of ROS

**Figure 8:**
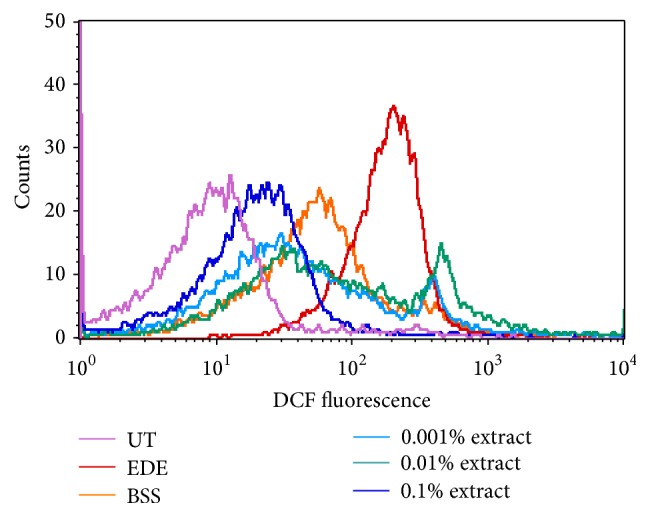
Levels of extracellular reactive oxygen species (ROS) measured using 2′,7′-dichlorodihydrofluorescein diacetate (DCFDA) with representative images of untreated control (UT), experimental dry eye (EDE), balanced salt solution (BSS), and 0.001%, 0.01%, and 0.1% extract-treated groups 10 days after desiccating stress.

The mean fluorescence intensity increased in the EDE group (195.38 ± 31.84) compared with the UT group (12.50 ± 2.93) 10 days after exposure to desiccating stress. The mean fluorescence intensities were 164.38 ± 14.02, 163.88 ± 23.17, 151.89 ± 22.60, and 58.13 ± 10.09 in the BSS and 0.001%, 0.01%, and 0.1% extract-treated groups, respectively. The 0.1% extract-treated group showed a significant decrease in the mean fluorescence intensities compared to the EDE and BSS-treated groups (*P* < 0.01) (Figure 8).

## 4. Discussion

Extensive research studies during the past decade have demonstrated the mechanisms by which imbalances in the redox status of prooxidant/antioxidant reactions can cause peroxidation of nucleic acids, bases, lipids, proteins, and carbohydrates and thereby cause ocular surface diseases including DED [33]. In addition, numerous antioxidants have shown beneficial effects on DED in the human corneal epithelial cell, as well as in murine dry eye animal models, and dry eye patients [21–26, 28, 34–36].

The components of the medicinal plant mixtures used in this study are known to have antioxidant and anti-inflammatory properties [37–40]. Our previous study established the antioxidant effects of a mixture of these natural plant EtOH extracts and their protection of human corneal epithelial cells from oxidative stress induced by short wavelength LED irradiation [28]. In this investigation, increased mRNA and protein expression of the antioxidant enzymes heme oxygenase-1, peroxiredoxin-1, catalase, and superoxide dismutase-2 in corneal epithelial cells were observed by the medicinal plant EtOH extracts in a dose-dependent manner. Additionally, the extracts of medicinal plants suppressed the amount of ROS generated by LED irradiation [28]. We also demonstrated that the extract mixture had a higher half-maximal-inhibitory concentration (IC_50_) than each of the individual EtOH extracts [28]. Additionally, in the prospective, multicenter, double-blind, randomized, placebo-controlled trial, we used glasses containing identical extracts of the medicinal plants to demonstrate their antioxidant effects in patients with mild DED. The ocular surface disease index score and tear film BUT significantly improved in the treatment group 4 and 8 weeks after wearing the glasses [29]. In the aspects of the results obtained in our prior studies, oxidative stress sustained the inflammation and apoptotic cell death and, therefore, we hypothesized that the use of antioxidant topical extracts in ophthalmic preparations could also be beneficial in the treatment of DED. Therefore, we evaluated therapeutic efficacy of extracts from antioxidant plants through topical application of various concentrations for treating DED by evaluating their improvement of various clinical parameters as well as inflammatory molecules and oxidative stress markers in a murine model of EDE.

Despite the continuous exposure to desiccating stress and rigorous anticholinergic treatment, the various clinical parameters including tear volume, tear film BUT, and corneal fluorescein staining scores showed clinical improvements on day 10 in the 0.1% extract-treated group compared with EDE and BSS-treated groups. In addition, the 0.01% extract-treated group showed increased tear production 10 days after the induction of desiccating stress compared to the EDE and BSS-treated groups.

The results of the multiplex immunobead assay indicated that the eye drops containing 0.1% medicinal plant extract reduced the inflammation of the ocular surface. The conjunctiva of the 0.1% extract-treated group showed decreased expressions of MIG, IL-1*β*, IL-6, IFN-*γ*, TNF-*α*, and IP-10 compared with EDE and BSS-treated groups 10 days after desiccating stress. Our present findings are supported by the results of recent studies showing that topical antioxidant application also had anti-inflammatory activity in murine dry eye model. Li et al. [24] and Rashid et al. [36] reported that topical omega-3 essential fatty acids (EFAs) decreased inflammatory markers such as CD11b+, IL-1*α*, IL-1*β*, IL-10, and TNF-*α* at both cellular and molecular levels and improved the corneal irregularity and staining scores of the murine dry eye model.

The homing and infiltrating T cells on the ocular surface are predominantly CD4+ T cells in DED [41, 42]. Furthermore, Th-1-related chemokine receptors such as the CXCR3 and their ligands have a pivotal role in the infiltration of activated CD4+ T cells [43, 44]. In this study, the conjunctiva of the 0.1% extract-treated group showed decreased CD4+CXCR3+ expression compared to the EDE and BSS-treated groups. Our results proved that the topical application of antioxidant extracts has a beneficial effect by effectively decreasing CD4+CXCR3+ T cell infiltration on the ocular surface.

In the present study, the 0.01% and 0.1% extract-treated groups achieved higher goblet cell densities than the EDE and BSS-treated groups. It is known that conjunctival goblet cells can be decreased by inflammation of the ocular surface while IL-6 and IFN-*γ* promote the loss of goblet cells from the conjunctival epithelium in desiccating stress [35, 45]. The degree of CD4+ T cell infiltration was inversely correlated with the conjunctival goblet cell density in desiccating stress-induced dry eye [46]. These results indicate that decreased inflammation of the conjunctival epithelium induced by the topical administration of antioxidant medicinal plant extracts might improve the goblet cell density.

4-HNE is a major end product of the oxidation of polyunsaturated fatty acids and is frequently measured as an indicator of lipid peroxidation and oxidative stress. Lipid peroxides and their breakdown products directly or indirectly affect numerous functions that are critical to cellular and organ homeostasis [47–49]. In patients with DED, the levels of 4-HNE in the tear and conjunctiva were observed to be significantly higher than those in the normal controls [11, 14]. In agreement with the results of previous reports, we showed that desiccating stress-stimulated production of 4-HNE was improved by the topical application of the antioxidant medicinal plant extract compared to the EDE and BSS-treated group 10 days after induction of EDE.

Furthermore, the extracellular ROS assay using confocal microscopy revealed that the fluorescence intensity was notably increased in the EDE group compared to the UT group while it was attenuated by topical application of 0.1% extract compared with the EDE and BSS-treated groups. These findings suggest that the topical application of medicinal plant extracts protected the ocular surface from desiccating stress in a murine dry eye model via antioxidative defense mechanisms.

The present study was conducted only with EtOH extracts of a mixture of various medicinal plants. However, additional studies with fractionated extracts should be performed to elucidate the active principles of the medicinal plant mixture. Furthermore, studies are also needed to compare the antioxidative activities of other identified antioxidants.

In summary, although the detailed mechanisms remain to be clarified, this study confirmed that the topical administration of antioxidant medicinal plant extracts improved the clinical parameters, suppressed the expression of inflammatory markers, attenuated oxidative damage, and reduced ROS generation in the ocular surfaces of mice exposed to desiccating stress by protecting against oxidative stress and inflammation. Therefore, the topical application of the extracts of antioxidant medicinal plants can be an alternative treatment option for treating the patients with DED.
